# Baseline cardiac output and its alterations during ibuprofen treatment for patent ductus arteriosus in preterm infants

**DOI:** 10.1186/s12887-019-1560-1

**Published:** 2019-06-05

**Authors:** Kai-Hsiang Hsu, Tai-Wei Wu, I-Hsyuan Wu, Mei-Yin Lai, Shih-Yun Hsu, Hsiao-Wen Huang, Tze-Yee Mok, Cheng-Chung Lee, Reyin Lien

**Affiliations:** 1 Division of Neonatology, Department of Pediatrics, Chang Gung Memorial Hospital Linkou Branch, Taoyuan, Taiwan; 2 Graduate Institute of Clinical Medical Science, Chang Gung University, Taoyuan, Taiwan; 3 Center for Fetal and Neonatal Medicine, Division of Neonatology, Children’s Hospital Los Angeles and Keck School of Medicine, University of Southern California, Los Angeles, CA USA; 4 Division of Neonatology, Department of Pediatrics, Chang Gung Memorial Hospital Keelung Branch, Keelung, Taiwan

**Keywords:** Cardiac output, Electrical cardiometry, Hemodynamic, Non-invasive monitor, Patent ductus arteriosus, Preterm infant

## Abstract

**Background:**

Infants with hemodynamically significant patent ductus arteriosus (PDA) may physiologically compensate with a supranormal cardiac output (CO). As such, a supranormal CO may be a surrogate marker for a significant PDA or indicate a failed response to PDA closure by ibuprofen. Electrical cardiometry (EC) is an impedance-based monitor that can continuously and non-invasively assess CO (CO_EC_). We aimed to trend CO_EC_ through ibuprofen treatment for PDA in preterm infants.

**Methods:**

We reviewed our database of preterm infants receiving ibuprofen for PDA closure. Response to ibuprofen was defined as no ductal flow in echocardiography ≤24 h after treatment. Responders were compared with gestational age (GA) and postnatal age matched non-responders and their trends of CO_EC_ were compared. Both groups’ baseline CO_EC_ were further compared to the reference infants without PDA.

**Results:**

Eighteen infants (9 responders and 9 non-responders) with median (interquatile range) GA 27.5 (26.6–28.6) weeks, birthweight 1038 (854–1218) g and age 3.5 (3.0–4.0) days were studied. There were positive correlations between CO_EC_ and ductal diameter and left atrium/ aortic root ratio (r = 0.521 and 0.374, *p* < 0.001, respectively). Both responders and non-responders had significantly higher baseline CO_EC_ than the reference. Although there was no significant within-subject alteration of CO_EC_ during ibuprofen treatment, there was a between-subject difference indicating non-responders had generally higher CO_EC_.

**Conclusions:**

The changes of CO_EC_ during pharmacological closure of PDA is less drastic compared to surgical closure. Infants with PDA had higher baseline CO_EC_ compared to those without PDA, and non-responders had higher CO_EC_ especially at baseline compared to responders.

## Introduction

Patent ductus arteriosus (PDA) is common among preterm infants and failure of ductal closure is associated with complications and poor outcomes [1]. Non-selective cyclooxygenase (COX) inhibitor, such as ibuprofen, is the pharmacological choice of treatment for PDA based on its action of prostaglandin inhibition that promotes ductal constriction. Both the intravenous and oral routes of ibuprofen administration appear comparably effective for ductal closure [2]. However, successful PDA closure by pharmacological treatment is not always definite or predictable [3]. The rate of ductal closure after COX inhibitors varies from 60 to 85% in preterm infants and is even less effective in extremely premature infants [4–6].

Echocardiography is often used to evaluate hemodynamic significance of PDA [7]. In general, pharmacological closure of PDA is less successful in infants with ductal diameter > 2 mm [8]. Lower ductal maximum velocity, which is usually associated with a larger PDA or higher pulmonary pressure, is another predictor of treatment failure [4, 8]. Furthermore, an increase in left ventricular cardiac output (CO) has been positively correlated with significant ductal shunting [7, 9, 10] and PDA severity [11]. The underlying reason is that a PDA with significant left-to-right flow may lead to a compensatory increase in CO in order to maintain systemic blood flow [12, 13]. Indeed, following closure of ductus after COX inhibitor therapy [12] or surgical ligation [10, 14], CO normalizes accordingly. We therefore hypothesized that a supranormal CO in the first week of life in extreme premature infants may indicate a hemodynamically significant PDA and that we could observe CO changes during pharmacological treatment. However, the ability to perform neonatal functional echocardiography requires practice, training and mentorship [15]. Furthermore, the use of echocardiography to gather meaningful hemodynamic data often necessitates serial assessments that can be tedious and labor-intensive.

Electrical cardiometry (EC) is a non-invasive, impedance-based monitor that provides absolute CO estimates in clinical practice [16]. Unlike echocardiography, EC is simple to apply***,*** continuous in measurements and not operator-dependent***.*** Comparisons between CO measured by EC (CO_EC_) and echocardiography have been studied in term [17] and preterm [18–20] infants with and without PDA. Although CO values measured by EC and echocardiography may not be interchangeable, it has been suggested that EC can be useful in trending CO changes in the clinical setting [20]. Hemodynamic reference by EC for neonates without PDA and without invasive ventilation support has been established, and CO_EC_ is positively correlated with gestational age (GA) and weight [21]. In addition, EC was used to monitor the effects of ductal ligation on CO_EC_, which revealed an initial decline in CO_EC_ followed by recovery [22]. Utilizing the ability of EC to continuously measure CO_EC_, we aimed to identify significant changes in CO_EC_ during attempted pharmacological closure and compared CO_EC_ characteristics in responders versus non-responders.

## Methods

### Patients

This study was conducted in the neonatal intensive care unit of Chang Gung Memorial Hospital Linkou Branch and was approved by the Institutional Review Board. As part of a hemodynamic monitoring project in the unit, echocardiographic findings and relevant hemodynamic information were collected prospectively into a database. We reviewed this database for very low birth weight (VLBW, < 1500 g) preterm infants admitted between June 2015 to June 2016 who received ibuprofen treatment for PDA closure. We enrolled infants who had both echocardiography and EC data during the first treatment course. Infants with chromosomal anomaly or structural heart defect other than small patent foramen ovale or atrial septal defect were excluded. Demographic data, serial echocardiographic findings and respiratory support at time of ibuprofen administration were collected.

### Ibuprofen for PDA closure

The decision to initiate ibuprofen for PDA closure was made based on individual’s clinical condition (e.g. increased respiratory support or hypotension) and echocardiographic finding (e.g. large ductus > 2 mm or low peak systolic ductal flow). Per unit policy, infants with right-to-left or bidirectional shunting PDA, intraventricular hemorrhage grade ≥ 3 or poor renal function (serum creatinine > 1.8 mg/dl or oligouria < 1 ml/kg/hr) were not candidates for ibuprofen treatment. The decision to treat with oral (ibuprofen oral suspension, [Center Laboratories Inc., Taipei, Taiwan]) or intravenous ibuprofen (Ibusine: Ibuprofen Lysine, [China Chemical & Pharmaceutical Co., Taipei, Taiwan]) was also made by the attending neonatologist. One course of treatment for both oral and intravenous ibuprofen consisted of three consecutive doses of 10, 5, 5 mg/kg/dose given 24 h apart. Responder to ibuprofen treatment was defined as absence of ductal flow in echocardiography within 24 h after completion of treatment.

### Echocardiography

Transthoracic echocardiography was performed using Sonos 7500 (Philips, Andover, Massachusetts, USA) with a 12 MHz transducers. Serial echocardiography was performed in relation to ibuprofen administration: within an hour prior to dose #1 ibuprofen (baseline), 18–24 h after dose #1 and #2 (during treatment), and 24 h after dose #3 of ibuprofen (treatment completion). This timeframe was chosen to allow maximum effect of each dose. Echocardiographic parameters of the PDA were assessed, which includes ductal size and shunt direction by color Doppler mapping, maximum flow velocity by pulsed-wave Doppler, and left atrium to aortic root ratio (LA/Ao) and left ventricular fractional shortening (FS) by M-mode.

### Electrical Cardiometry (EC)

EC (Aesculon, Osypka Medical, Berlin, Germany) was applied by attaching four standard surface electrocardiogram electrodes over the forehead, left lower neck, left mid-axillary line at the level of xiphoid process and lateral aspect of left thigh. EC was placed at least 1 h prior to dose #1 ibuprofen and kept in situ until 24 h after completing treatment. Hemodynamic parameters by EC, including CO_EC_, heart rate (HR_EC_) and stroke volume (SV_EC_) were captured every 10 min during the study period and subsequently exported into a database using software Waveform Explorer by Osypka Medical. The original data that 1 h before treatment and 18–24 h after each ibuprofen dose were further averaged and analyzed (e.g. the baseline and 18–24 h following dose #1, #2 and #3, respectively). Value of CO_EC_ and SV_EC_ were weight-adjusted as ml/kg/min and ml/kg.

### Matching

In order to minimize confounders related to GA, weight and post-natal age, we matched each responder to a non-responder with comparable GA ± 1 week, weight ± 10% g and post-natal age ± 7 days from the hemodynamic database. Furthermore, for comparison of CO_EC_ between infants with and without PDA, we also matched above responders and non-responders respectively to our previously published reference [21] using the same criteria.

### Statistics

Statistical analysis was performed using IBM SPSS Statistics version 20 (Armonk, NY, USA). Continuous variables in background demographics were tested using Mann-Whitney U test, while hemodynamic parameters by EC were tested with independent *t*-test between responders and non-responders or paired *t*-test was between two timing points. Repeated measures analysis of variance (RM-ANOVA) was applied to compare trends of hemodynamic parameters through the course. Categorical data were analyzed with Chi-square test or Fisher’s exact test. Analysis of the relationship between CO_EC_ and ductal diameter or LA/Ao was by Pearson correlation coefficient. One-way ANOVA with Bonferroni correction was used to compare CO_EC_ among responders, non-responders and the reference. Statistical significance was defined as two-sided *p* < 0.05.

## Results

**Fig. 1:**
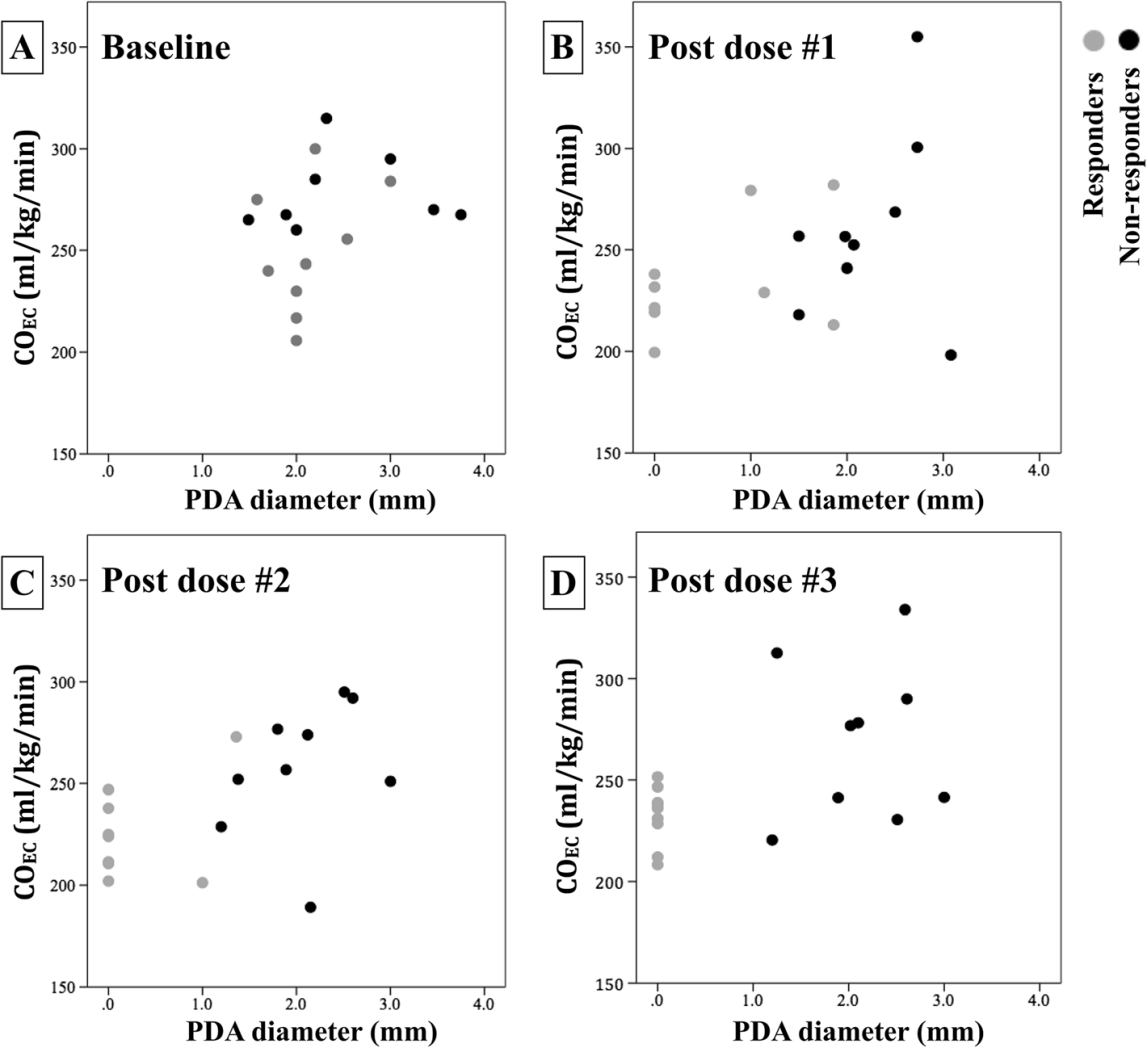
Scatter diagrams of CO_EC_ and ductal diameter for preterm infants who responded (gray circles) and non-responded (black circles) to ibuprofen treatment for PDA. Four timing points were plotted: 1 h prior to treatment (baseline, **a**) and 18–24 h post each dosage of ibuprofen (**b**, **c** and **d**, respectively). CO_EC_, cardiac output by electrical cardiometry; PDA, patent ductus arteriosus

During the study period, 303 VLBW preterm infants were admitted to our unit, of which 46 received ibuprofen treatment. There was complete data collection for both echocardiography and EC in 36 infants, and 11 of them were responders. After screening and matching, 9 out of 11 responders could be matched to 9 non-responders, and a total of 18 preterm infants were included. Their median (interquartile range) GA, weight and post-natal age at initiation of ibuprofen were 27.5 (26.6–28.6) weeks, 1038 (854–1218) g and 3.5 (3.0–4.0) days old, respectively. There was no significant difference in demographics, echocardiographic measurements, post-natal age, route of ibuprofen or respiratory support between responders and non-responders (Table 1). None received vasopressor or inotrope during the treatment course. Among 9 responders, 5 infants were found to have absence of ductal flow after dose #1 ibuprofen, 2 infants after dose #2, and 2 infants after dose #3 (Fig. 1 a–d). Furthermore, there was positive correlations between CO_EC_ and ductal diameter (*r* = 0.521, *p* < 0.001) and LA/Ao (*r* = 0.374, *p* < 0.001).

**Table 1:** Clinical characteristics for responders and non-responders for ibuprofen treatment for PDA

*Demographics*	Responders	Non-Responders	*p* value^a^
	(*n* = 9)	(*n* = 9)	
Gestational age (weeks)	27.7 (27.1–29.9)	27.4 (26.1–27.9)	0.161
Weight (g)	1135 (913–1318)	1015 (830–1083)	0.222
Apgar at 1 min	7 (5–8)	6 (3–7)	0.077
Apgar at 5 min	9 (7–9)	9 (8–9)	0.931
Male	6 (67%)	2 (22%)	0.153
Cesarean section	6 (67%)	7 (78%)	1.000
Small for gestational age	2 (22%)	1 (11%)	1.000
*Echocardiography prior to ibuprofen treatment*			
PDA diameter (mm)	2.05 (1.78–2.46)	2.20 (1.70–3.23)	0.666
PDA diameter to weight (mm/kg)	2.04 (1.40–2.54)	2.26 (1.56–3.57)	0.340
PDA maximum flow velocity (m/s)	2.21 (1.59–2.60)	1.82 (1.30–2.52)	0.613
LA/Ao ratio	1.47 (1.27–1.76)	1.50 (1.44–1.87)	0.370
Fractional shortening (%)	41.0 (36.0–44.5)	39.0 (34.2–43.8)	0.661
*Condition prior to ibuprofen treatment*			
Post-natal age at dose #1 (day)	3.0 (3.0–4.0)	4.0 (3.0–6.5)	0.222
Oral ibuprofen	8 (89%)	7 (78%)	1.000
Respiratory support			0.183
Non-invasive ventilation	5 (56%)	2 (22%)	
Conventional ventilation	4 (44%)	5 (56%)	
High frequency ventilation	0 (0%)	2 (22%)	

*PDA* patent ductus arteriosus, *LA/Ao* left atrium to aortic root diameter

Data are median (interquartile range) or n (%)

^a^A *p* value was tested by Mann-Whitney U test for continuous variables and Chi-square test or Fisher’s exact test for categorical data

**Fig. 2:**
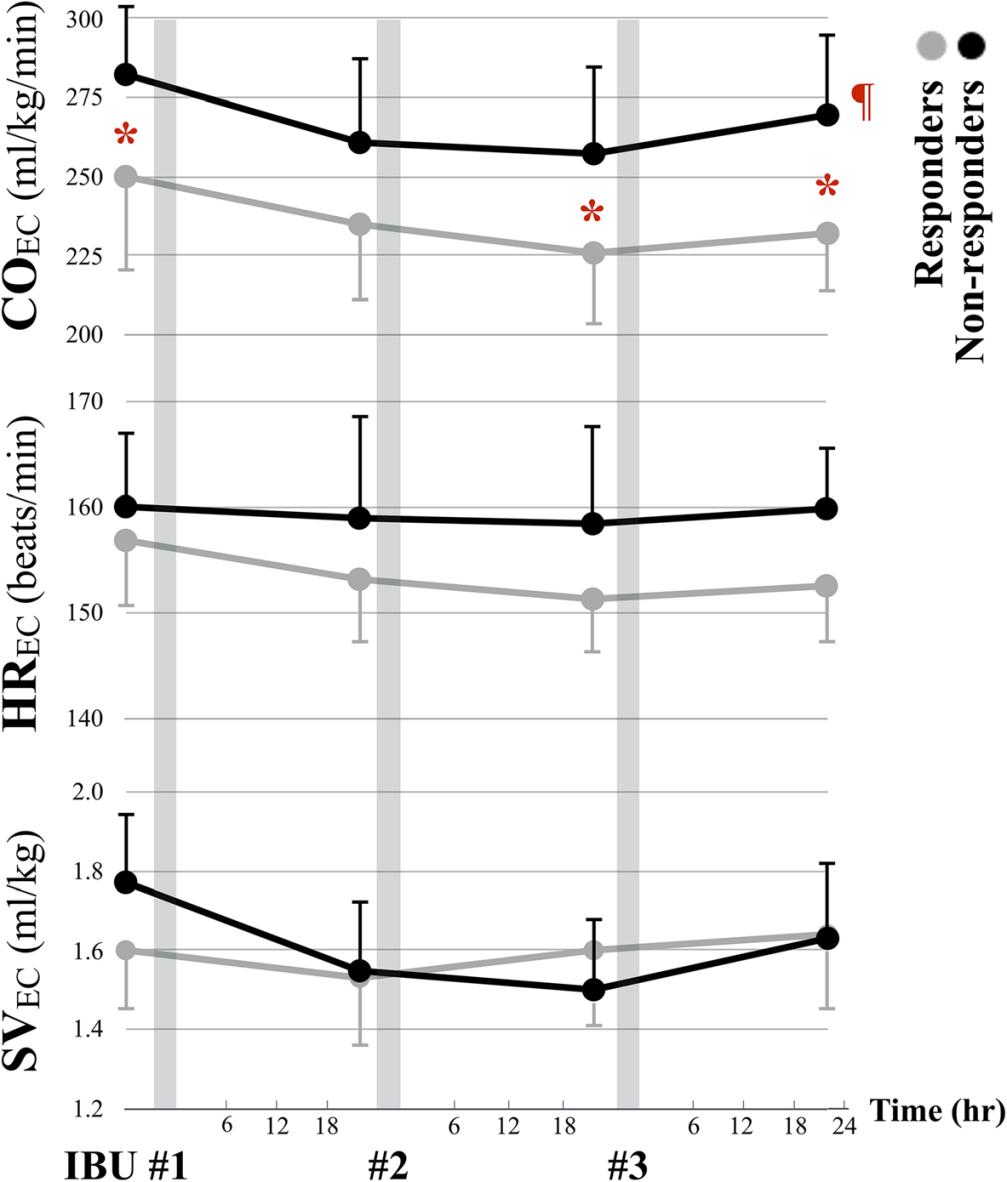
Trends charts of CO_EC_, HR_EC_ and SV_EC_ for responders (gray line) and non-responders (black line) through ibuprofen treatment. Three gray bands indicate the time of each ibuprofen administration. Although there was no remarkable alteration of CO_EC_, HR_EC_ and SV_EC_ within each group, non-responders had significantly higher CO_EC_ than responders through the course (between-subject *p* = 0.005) (¶), especially at the timing prior to dose #1 ibuprofen, 18–24 h post dose #2 and 18–24 h post dose #3, respectively (*). CO_EC_, cardiac output; HR_EC_, heart rate; SV_EC_, stroke volume; all measured by electrical cardiometry

Non-responders had higher CO_EC_ compared to responders throughout the treatment course (RM-ANOVA between-subject *p* = 0.005). This discrepancy was most significant prior to ibuprofen treatment (282 ± 21 vs. 250 ± 31 ml/kg/min, *p* = 0.022), at 24 h post dose #2 (257 ± 33 vs. 226 ± 23 ml/kg/min, *p* = 0.034), and 24 h post dose #3 (270 ± 39 vs. 232 ± 14 ml/kg/min, p = 0.022) (Fig. 2a). No significant differences in HR_EC_ or SV_EC_ were found between the two groups (Fig. 2b and c).

When analyzing within-subject changes throughout the treatment course, there were no significant changes of CO_EC_, HR_EC_ or SV_EC_ in either responders or non-responders (RM-ANOVA). The average alteration of CO_EC_ was − 7% ± 12% for responders and − 6% ± 16% for non-responders. On the other hand, when comparing baseline CO_EC_ to the earliest time point when no ductal flow was visualized by echocardiography, there was a significant but small-scale reduction in CO_EC_ by 25 ml/kg/min or 10% (250 ± 31 vs. 225 ± 17 ml/kg/min, paired *t*-test *p* = 0.031) (Table 2). However, we found 4/9 (44%) of non-responders had > 10% reduction of CO_EC_ at some timing points as well.

**Table 2:** Hemodynamic changes at specific timing points

		Responders (*n* = 9)	Non-responders (*n* = 9)	*p* value
CO_EC_ (ml/kg/min)	Prior to dose #1	250 ± 31	282 ± 21	0.022^b^
	No ductal flow^a^	225 ± 17^c^	N/A	N/A
	18–24 h after dose #3	232 ± 15	270 ± 39	0.021^b^
HR_EC_ (beats/min)	Prior to dose #1	157 ± 7	160 ± 8	0.394
	No ductal flow^a^	151 ± 7	N/A	N/A
	18–24 h after dose #3	153 ± 8	160 ± 6	0.077
SV_EC_ (ml/kg)	Prior to dose #1	1.59 ± 0.23	1.77 ± 0.30	0.165
	No ductal flow^a^	1.50 ± 0.15	N/A	N/A
	18–24 h after dose #3	1.63 ± 0.29	1.63 ± 0.25	0.926

*CO* cardiac output, *HR* heart rate, *SV* stroke volume, *EC* electrical cardiometry, *N/A* not applicable

Data are mean (± SD)

^a^Five infants’ ductal flow disappeared in color Doppler post dose #1, two post dose #2 and two post dose #3

^b^indicates statistical significance between responders and non-responders (independent t-test)

^c^indicates statistical significance comparing to baseline value prior to dose #1 (paired t-test)

**Fig. 3:**
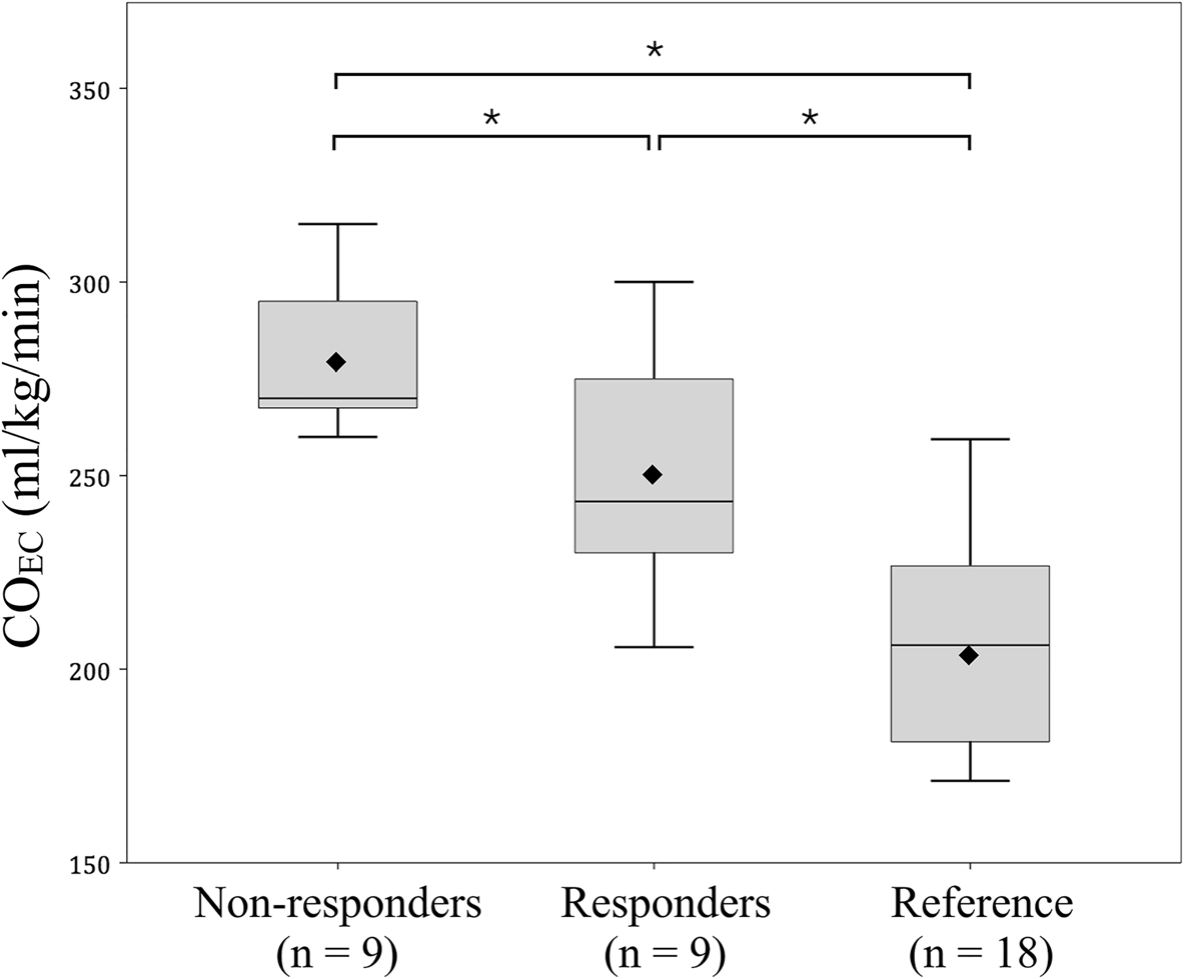
Box plot of baseline CO_EC_ for responders, non-responders and matched reference. The horizontal lines are median CO_EC_ and the diamond marks are mean of CO_EC_ for respective group. Mean CO_EC_ of three groups were statistically different, especially non-responders had the highest CO_EC_. CO_EC_, cardiac output by electrical cardiometry

Another 18 infants without PDA were matched for baseline CO_EC_ comparison. Their median GA and weight were 28.6 (28.0–30.2) weeks and 1175 (1005–1312) g, respectively, and were all 3–4 days old. No demographic difference existed among these three groups (responders, non-responders and the reference). There was a significant stepwise increment in baseline CO_EC_ from infants with no PDA (207 ± 28 ml/kg/min), to infants with PDA, responders (250 ± 31 ml/kg/min), to infants with PDA, non-responders (282 ± 21 ml/kg/min, *p* < 0.001) (Fig. 3).

## Discussions

In this study, we showed the potential of EC to continuously monitor changes in CO_EC_ among preterm infants. By carefully matching target infants, we demonstrated that infants with PDA had higher baseline CO_EC_ and there was no significant CO_EC_ alteration during ibuprofen treatment for ductal closure.

Our finding indicated that preterm infants with PDA have significantly higher baseline CO_EC_ compared to age-matched reference, and that baseline CO_EC_ is positively correlated to PDA diameter and LA/Ao. The positive correlation suggests that infants with greater CO_EC_ have a higher likelihood of more significant ductal shunting. It was interesting to find that only the baseline CO_EC_, but not ductal diameter, maximum ductal flow or LA/Ao, was significantly different between responders and non-responders in our study. It can be reasoned that with high left-to-right ductal shunting, CO represents the sum of systemic flow plus ductal shunting, and hence increases in CO is a compensation and proportional to ductal shunting [7, 13]. Furthermore, only CO_EC_ but not HR_EC_ or SV_EC_ was significantly different between responders and non-responders. This may indicate that CO represents the sum of left ventricular work, i.e., HR and SV, to compensate for the ductal steal effect. It also suggests that CO may be a more comprehensive surrogate in determining the degree of ductal shunting. The difference in baseline CO_EC_ between responders and non-responders is compatible with previous studies that infants with larger ductal shunting may response poorly to COX inhibitor [4, 8].

We observed no significant CO_EC_ alteration through ibuprofen treatment for PDA closure. Although there was a mean decrease of CO_EC_ by 10% on initial ductal closure, this reduction of CO_EC_ cannot be an indicator for ductal closure because non-responders may also had > 10% reduction of CO_EC_ through the course. Moreover, the small-scale decline is unlike our previous study that a 26% decrease in CO_EC_ at time of ductal ligation [22]. We speculate that the effect of ibuprofen in inducing ductal closure was progressive or intermittent while allowing time for the myocardium to adapt to the hemodynamic changes. This is further supported by the fact that no infant in our study required inotropic support, which is needed in infants with post-ligation hemodynamic instability.

There is a similar study utilizing EC to monitor CO during attempted pharmacological closure of PDA by intravenous ibuprofen in preterm infants [23], of which, a fall in median CO_EC_ from 290 to 240 ml/kg/min (17%) 72 h after the initiation of treatment was found. However, the study is limited by its small case number (6 responders) and a wide overlap of CO_EC_ between baseline and 72 h after the first dose ibuprofen. In addition, 2 out of 6 infants in this study received dopamine infusion before ibuprofen treatment and dopamine was tapered off at the end of ibuprofen treatment, which can confound the baseline and post-treatment CO_EC_ measurements [24]. The dopamine infusion may have contributed to the larger discrepancy between baseline and post-treatment CO_EC_ in this study.

Some limitations should be addressed. Firstly, the sample size of current study was small. The number of responders limited the power to demonstrate exact CO_EC_ changes and to detect a confident cut-off CO_EC_ to assess treatment response. Secondly, using echocardiography to detect the exact timing of ductal closure during ibuprofen treatment is clinically complex. We are only able to use the earliest available echocardiography data that indicates no ductal flow to assess CO_EC_ alteration. This also limited the ability to estimate short-term alteration following ductal closure. We also lacked other echocardiographic markers for PDA severity such as superior vena cava flow for systemic blood flow [25] or left pulmonary artery end-diastolic flow for pulmonary overcirculation [26]. Thirdly, some demographic information was not included into analysis. Closure of PDA is a multifactorial interaction, complete respiratory evaluation inclusive of arterial blood gas analysis, inhaled oxygen fraction and mean airway pressure, and even genetic disposition or pharmacokinetic difference should be considered. Lastly, we merely analyzed infants who received the first treatment course. Since it is known that the ibuprofen response is accumulative, it is warranted to enroll those receiving repeated courses in a future study.

## Conclusions

The decrease in CO_EC_ during pharmacological closure of PDA is less drastic. Baseline CO measured by EC is higher in infants with PDA compared to those without PDA, especially non-responders had higher CO_EC_ at baseline compared to responders. Monitoring CO_EC_ is clinically applicable in bedside hemodynamic trending; however, a detailed assessment of hemodynamic compensation to a significant ductal shunt and to estimate pharmacological closure of the duct requires further studies.

## Data Availability

The dataset supporting the conclusions of this article is available by inquiring to khsu@cgmh.org.tw.

## Abbreviations

CO: Cardiac output
COX: Cyclooxygenase
EC: Electrical cardiometry
HR: Heart rate
PDA: Patent ductus arteriosus
RM-ANOVA: Repeated measures analysis of variance
SV: Stroke volume
VLBW: Very low birth weight
